# Transcriptomic and Metabolomic Insights into Plant Hormone Modulation and Secondary Metabolite Accumulation in Basil Under Far-Red and Ultraviolet-A Light

**DOI:** 10.3390/ijms26083758

**Published:** 2025-04-16

**Authors:** Dandan Li, Haibin Shen, Lishu Yu, Kaili Zhu, Yongxue Zhang, Shaofang Wu, Liying Chang, Xiaotao Ding, Yuping Jiang

**Affiliations:** 1 College of Ecological Technology and Engineering, Shanghai Institute of Technology, Shanghai 201418, China; lddwyzh2022@163.com (D.L.); 15921457938@163.com (L.Y.); z18253926832@163.com (K.Z.); 2 Shanghai Key Laboratory of Protected Horticulture Technology, Horticultural Research Institute, Shanghai Academy of Agricultural Science, Shanghai 201403, China; shb8311@163.com (H.S.); xuezylemon@foxmail.com (Y.Z.); sfwu@saas.sh.cn (S.W.); 3 School of Agriculture and Biology, Shanghai Jiao Tong University, Shanghai 200240, China; changly@sjtu.edu.cn

**Keywords:** flavonoid biosynthesis, FR light, GA, JA, UVA light

## Abstract

In this study, red–blue light (7R3B) was used as the control (CK), while far-red (FR) and ultraviolet-A (UVA) light were supplemented to evaluate their effects on basil growth. The results showed that the FR treatment promoted plant height, stem diameter, and biomass, but reduced chlorophyll and carotenoid content, while the UVA treatment increased stem diameter and chlorophyll b content. Meanwhile, transcriptomic and metabolomic analyses were employed to examine changes in gene expression and metabolite accumulation in basil. The FR treatment reduced the levels of differentially accumulated metabolites (DAMs) in the carotenoid biosynthesis pathway, potentially contributing to the observed decrease in chlorophyll. The FR treatment upregulated the levels of five DAMs (gibberellin, cytokinin, brassinosteroid, jasmonic acid, and salicylic acid) and altered the differentially expressed genes (DEGs) such as *gibberellin receptor* (*GID1*) and *jasmonate ZIM domain-containing protein* (*JAZ*) in the plant hormone signal transduction pathway, thereby promoting plant growth and shade avoidance responses. The UVA treatment upregulated the *9-cis-epoxycarotenoid dioxygenase* (*NCED*) expression in the carotenoid biosynthesis pathway, possibly indirectly promoting flavonoid synthesis. In the flavonoid biosynthesis pathway, the UVA treatment also promoted flavonoid accumulation by upregulating DEGs including *flavonol synthase* (*FLS*), *anthocyanidin synthase* (*ANS*), *5-O-(4-coumaroyl)-D-quinate 3′-monooxygenase* (*CYP98A*), and *flavanone 7-O-glucoside 2″-O-beta-L-rhamnosyltransferase* (*C12RT1*), as well as increasing the levels of DAMs such as kaempferol, luteolin, apigenin, and leucopelargonidin. The accumulation of flavonoids improved antioxidant capacity and nutritional value in basil. Through a Kyoto Encyclopedia of Genes and Genomes (KEGG) pathway enrichment analysis, this study provided valuable insights into the molecular and metabolic mechanisms of the FR and UVA regulation of basil growth, providing guidance for optimizing supplementary lighting strategies in plant factories.

## 1. Introduction

Basil (*Ocimum basilicum* L.), also known by names such as Jiucengta, Xianghuazi, and Lanxiang, is an annual herbaceous plant in the Lamiaceae family and the genus *Ocimum*. It is not only abundant in essential oils but also contains high concentrations of anthocyanins, total phenolics, and flavonoids—compounds recognized as potent antioxidants [1,2]. These bioactive constituents effectively scavenge free radicals, suppress oxidative processes, and exhibit significant anti-inflammatory [3] and antimicrobial activities [4]. Consequently, basil holds substantial promise for a wide range of applications, including cosmetics, fragrances, consumer goods, and pharmaceutical and healthcare products [5,6]. In recent years, the market demand for basil has diversified significantly, with the price of edible ornamental varieties ranging from USD 3 to 4 per pot, highlighting its substantial economic potential. Moreover, owing to its compact size and short growth cycle, basil is particularly well suited for intensive agricultural systems such as plant factories [7]. This green plant thus represents an ideal candidate for the development and promotion of high-end ornamental vegetables.

Light, as one of the primary environmental factors influencing plant growth and development, is closely associated with seed germination, vegetative growth, reproductive development, and senescence [8]. Among these, far-red (FR) and ultraviolet (UV) light represent two extreme segments of the spectrum, each playing unique roles and exerting specific influences on plant growth. When plants are exposed to a low red/far-red (R/FR) light ratio, they exhibit a series of phenotypic changes collectively referred to as the “shade avoidance syndrome” (SAS) [9]. These changes include the elongation of internodes, petioles, and hypocotyls, enhanced apical dominance, early flowering, and upward leaf movement [9]. Such responses improve the individual competitiveness and adaptability of plants within densely populated communities [10]. In Chinese kale, supplemental FR light significantly increased plant height, stem diameter, and yield [11]. Under FR supplementation, total biomass increased by 9–16%, while mature tomato yield increased by 7–12% [12]. Light-activated phytochromes inactivate phytochrome-interacting factors (*PIFs*) by inhibiting their DNA-binding activity and promoting their phosphorylation, ubiquitination, and degradation [13,14]. Under a low R/FR ratio, reduced phytochrome activity leads to an increased accumulation of *PIF3*, *PIF4*, and *PIF5* in *Arabidopsis* [15], while also enhancing *PIF7* binding to target promoters. Co-target genes activated by *PIF4*, brassinosteroids (BR), auxin (IAA), and gibberellin (GA) contribute to cell elongation through processes such as cell wall synthesis and loosening [16]. In addition, a low R/FR ratio promotes the accumulation of the dephosphorylated form of *PIF7* in the nucleus, thereby activating downstream auxin biosynthetic genes and ultimately promoting the occurrence of SAS [17]. A study found that *PIF* accumulation in *Arabidopsis* leaves elevates IAA levels, which are subsequently transported to the stem, promoting stem elongation [18]. In summary, a low R/FR ratio primarily regulates plant growth and development through phytochromes, *PIFs*, and endogenous hormones such as IAA, GA, abscisic acid (ABA), and cytokinins (CTK) [19,20,21].

UV radiation is categorized as UVC, UVB, and UVA. UVC and most UVB are absorbed by stratospheric oxygen and the ozone layer, allowing only approximately 4% of UVB to reach the Earth’s surface. In contrast, UVA is less harmful, reaches the Earth’s surface in large amounts, and is a key photomorphogenic signal for plant growth and development [22]. To counteract damage from UV radiation, plants have evolved various mechanisms, including accumulating phenolic compounds in the leaf epidermis to absorb UV radiation, repairing UV-induced DNA damage, and producing antioxidants to scavenge peroxides and oxygen radicals. While many herbaceous plants lack effective UV protection mechanisms themselves, they can respond to high UV flux by stimulating flavonoid synthesis [23]. Research has shown that UV radiation significantly increases the carbon partitioning of flavonoids in polyphenols regardless of visible light intensity [24]. Additionally, UV radiation is an effective inducer of secondary metabolite accumulation, including classes such as flavonoids, phenolics, alkaloids, carotenoids, and terpenoids, in vegetables, herbs, and fruits [25,26]. Enhancing the accumulation of various valuable secondary metabolites through light induction may generate significant economic benefits for the food, healthcare, and related industries [27]. While substantial research has focused on the effects of UVB radiation [28], UVA, despite its photon abundance—10 to 100 times greater than UVB in solar radiation—has been less studied due to methodological challenges [29]. It is hoped that this experiment will help fill some of the gaps regarding the effects of UVA radiation on plants.

Current research on light formulas in plant factories primarily focuses on different light periods and varying red–blue light ratios, with relatively fewer studies on FR and UVA light treatments. A study using transcriptomic analysis found that UVA light treatment significantly increased the flavonol content and the expression of related enzyme genes in the hybrid progeny of *Ginkgo*, enhancing the flavonoid content in *Ginkgo* and improving its medicinal value [30]. Through transcriptomic and metabolomic analyses, another study found that the addition of FR light promoted changes in the expression of auxin-related genes in pumpkin, and significantly upregulated the levels of IAA biosynthetic precursors tryptophan and indole, providing evidence for the mechanism of FR light-mediated hypocotyl elongation [31]. Research specifically on basil in plant factories, as well as the effects of FR and UVA light on basil, remains particularly limited. In this study, we aimed to elucidate the effects of supplemental FR and UVA light on basil by assessing growth parameters, photosynthetic pigment content, plant hormone levels, and secondary metabolite accumulation. To achieve this, basil plants grown under these lighting conditions were systematically evaluated for growth performance and pigment content. Furthermore, integrated transcriptomic and metabolomic network analyses were conducted to identify key genes and metabolites associated with FR light-induced growth enhancement and UVA-stimulated secondary metabolite synthesis, with a particular emphasis on flavonoid biosynthesis.

## 2. Results

### 2.1. Effect of FR and UVA Treatments on the Growth Indicators of Basil

**Figure 1:**
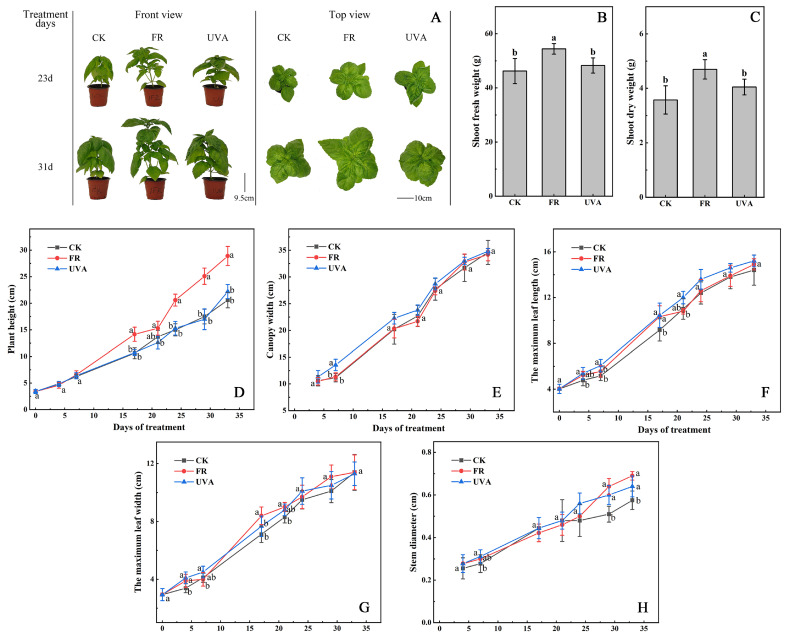
Analysis of phenotype and growth indexes of basil treated with FR and UVA light. CK: The red-to-blue light ratio is maintained at 7:3 (7R3B); FR: 7R3B + far red light; UVA: 7R3B + ultraviolet-A light. Different letters indicate significant differences at α = 0.05 based on the least significant difference (LSD) test (n = 3). (**A**) The photos of basil at 23 and 31 days under different light treatment conditions. (**B**) Shoot fresh weight. (**C**) Shoot dry weight. (**D**) Plant height. (**E**) Canopy width. (**F**) Maximum leaf length. (**G**) Maximum leaf width. (**H**) Stem diameter.

The morphology of basil exhibited significant differences under different treatments (Figure 1A). At the end of the experiment, the plant height and stem diameter of basil under the FR treatment were significantly greater than those of the control group (CK), indicating that the addition of FR light during the growth process significantly promoted stem growth (Figure 1D,H). The UVA treatment increased stem diameter but had no significant effect on plant height.

By regularly measuring the growth parameters of the plants, the impact of adding FR and UVA light on basil growth was assessed (Figure 1D–H). From day 7 of the experiment, basil under the FR treatment showed a gradual increase in plant height, surpassing both CK and UVA treatments, and was significantly higher by the end of the experiment (Figure 1D). The stem diameter under both the UVA and FR treatments was significantly larger than that of the CK treatment after 24 days of treatment (Figure 1H). Significant differences in canopy width, maximum leaf length, and maximum leaf width were observed during the early stages of the treatment (Figure 1E–G). However, as the experiment progressed, these differences between treatments gradually diminished, and no significant differences were observed by the end of the experiment.

Under the FR treatment, the shoot fresh weight was significantly higher than that of the CK treatment, increasing by 17.74%, and there was no significant difference between the UVA and CK treatments (Figure 1B). The shoot dry weight under the FR treatment was significantly higher than that of the CK treatment, increasing by 31.47%, and there was no significant difference between the CK and UVA treatments (Figure 1C).

### 2.2. Effects of FR and UVA Treatments on Chlorophyll and Carotenoid Content of Basil

Compared to the CK, the FR treatment significantly reduced the levels of chlorophyll a, chlorophyll b, carotenoids, and total chlorophyll by 10.7%, 11.8%, 13.5%, and 11%, respectively (Table 1). The UVA treatment significantly increased the basil chlorophyll b content by 2.8%.

### 2.3. Effects of FR and UVA Treatments on the Transcriptome of Basil

#### 2.3.1. Basil Transcriptome Sequencing Results

Transcriptome sequencing analysis of different treatments on basil samples yielded a total of 59.23 GB of clean data. The clean reads for each sample ranged from 20,747,666 to 23,428,741, with Q30 values ranging from 97.43% to 98.16% (Supplementary Materials: Table S1). Sequencing data were compared to the basil reference genome, with comparison efficiencies ranging from 95.79% to 96.51%, indicating reliable data acquisition suitable for subsequent analysis.

#### 2.3.2. The Gene Expression and Differentially Expressed Gene (DEG) Analysis of Basil Under FR and UVA Treatments

A total of 90,128 genes were detected, including 82,954 known genes and 7174 novel genes. A DEG analysis was conducted using a fold change ≥ 2 and FDR < 0.01 as the screening criteria, revealing 3073 and 2102 DEGs for FR vs. CK and UVA vs. CK treatments, respectively (Supplementary Materials: Figure S1). The DEG analysis revealed that in the FR vs. CK comparison, 1325 out of 3073 DEGs were upregulated, and 1748 were downregulated. In the UVA vs. CK comparison, 1277 out of 2102 DEGs were upregulated, and 825 were downregulated. The functional annotation of DEGs was performed using various databases (Supplementary Materials: Table S2), and the results indicated that the addition of FR and UVA had significant effects on the gene expression in basil.

#### 2.3.3. Kyoto Encyclopedia of Genes and Genomes (KEGG) Pathway Enrichment Analysis of Differentially Expressed Genes (DEGs) in Basil Under FR and UVA Treatments

**Figure 2:**
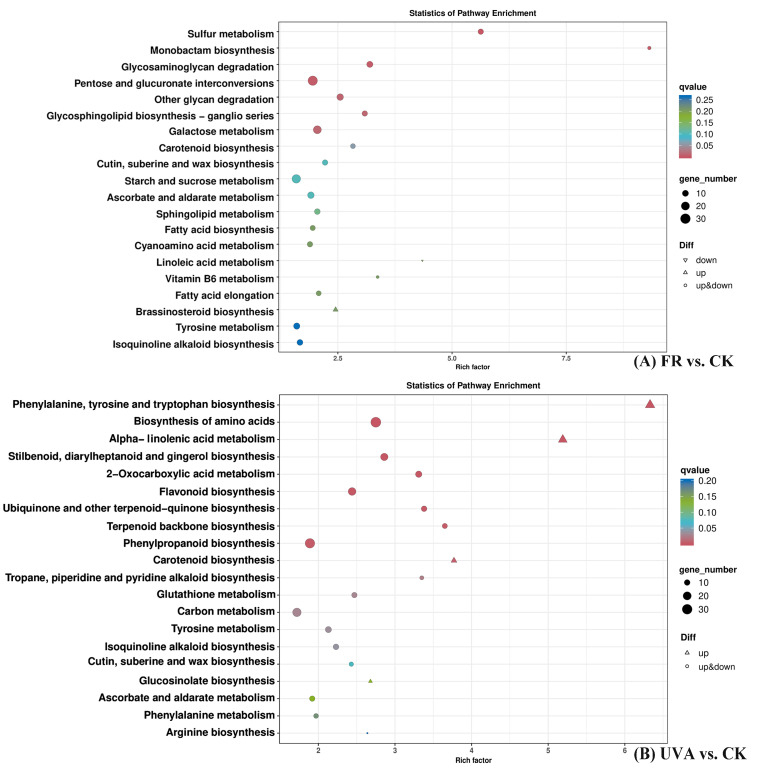
Kyoto Encyclopedia of Genes and Genomes (KEGG) pathway enrichment of differentially expressed genes (DEGs) between FR and CK treatments or UVA and CK treatments. CK: The red-to-blue light ratio is maintained at 7:3 (7R3B); FR: 7R3B + far red light; UVA: 7R3B + ultraviolet-A light.

Twenty KEGG pathways significantly enriched in DEGs in the FR vs. CK and UVA vs. CK treatment groups were selected, respectively (Figure 2). A pathway analysis revealed that in both the FR vs. CK and UVA vs. CK treatment groups, five pathways were notably enriched in DEGs, including carotenoid biosynthesis, cutin, suberin, and wax biosynthesis, ascorbate and aldarate metabolism, tyrosine metabolism, and isoquinoline alkaloid biosynthesis (Figure 2).

In the FR vs. CK treatment group (Figure 2A), the pathways with the most enriched DEGs were pentose and glucuronate interconversions, starch and sucrose metabolism, and galactose metabolism, with 34, 28, and 24 DEGs, respectively. The sulfur metabolism and monobactam biosynthesis pathways had the highest Rich factor values and the highest degree of DEG enrichment.

In the UVA vs. CK treatment group (Figure 2B), the pathways with the most enriched DEGs were the biosynthesis of amino acids, phenylpropanoid biosynthesis, and carbon metabolism, with 38, 35, and 28 DEGs, respectively. The phenylalanine, tyrosine, and tryptophan biosynthesis and alpha-linolenic acid metabolism pathways had the highest Rich factor values, at 6.33 and 5.19, respectively. The phenylalanine, tyrosine, and tryptophan biosynthesis and biosynthesis of amino acids pathways had the highest degree of DEG enrichment.

### 2.4. The Effects of FR and UVA Treatments on the Metabolome of Basil

#### 2.4.1. Metabolite Profiling and Differentially Accumulated Metabolite (DAM) Analysis of Basil Under FR and UVA Treatments

Between the CK and FR treatments, 1708 DAMs were identified, of which 918 were upregulated and 790 were downregulated (Supplementary Materials: Table S3). Similarly, in the comparison between the UVA and CK treatments, 1586 DAMs were detected, including 964 upregulated and 622 downregulated metabolites (Supplementary Materials: Table S3). The results showed that FR light and UVA light irradiation had a significant effect on the accumulation of basil metabolites.

#### 2.4.2. Kyoto Encyclopedia of Genes and Genomes (KEGG) Pathway Enrichment Analysis of Differentially Accumulated Metabolites (DAMs) in Basil Under FR and UVA Treatments

**Figure 3:**
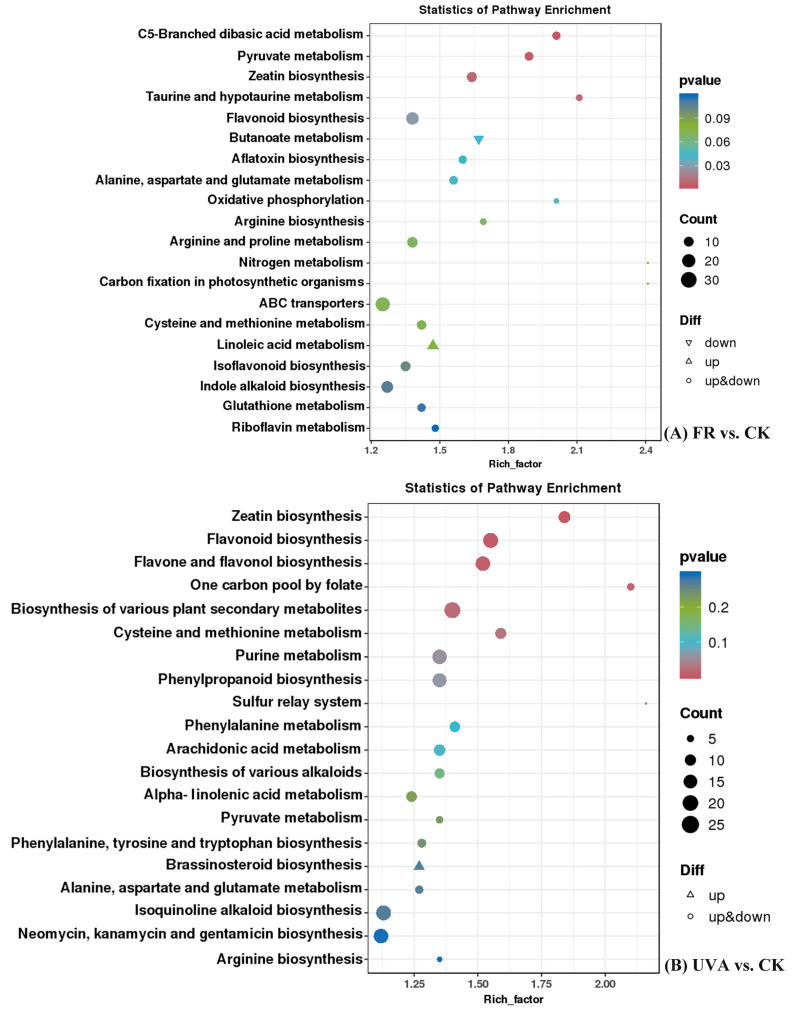
Kyoto Encyclopedia of Genes and Genomes (KEGG) pathway enrichment of differentially accumulated metabolites (DAMs) between FR and CK treatments or UVA and CK treatments. CK: The red-to-blue light ratio is maintained at 7:3 (7R3B); FR: 7R3B + far red light; UVA: 7R3B + ultraviolet-A light.

The 20 most significantly different KEGG metabolic pathways were identified in the FR vs. CK and UVA vs. CK treatment groups (Figure 3). In the FR vs. CK treatment group (Figure 3A), the most enriched pathways, both pyruvate metabolism and zeatin biosynthesis are associated with plant hormones, which indicates that the FR treatment significantly affected DAM levels related to plant hormones.

In the UVA vs. CK treatment group (Figure 3B), the pathways with the highest enrichment, flavonoid biosynthesis, and flavone and flavonol biosynthesis are both associated with flavonoid synthesis, indicating that the UVA treatment affected the synthesis of flavonoid compounds.

### 2.5. Combined Analysis of Transcriptome and Metabolites in Basil Under FR and UVA Treatments

#### 2.5.1. Combined Analysis of Transcriptome and Metabolites in Carotenoid Biosynthetic Pathway Under FR and UVA Treatments

**Figure 4:**
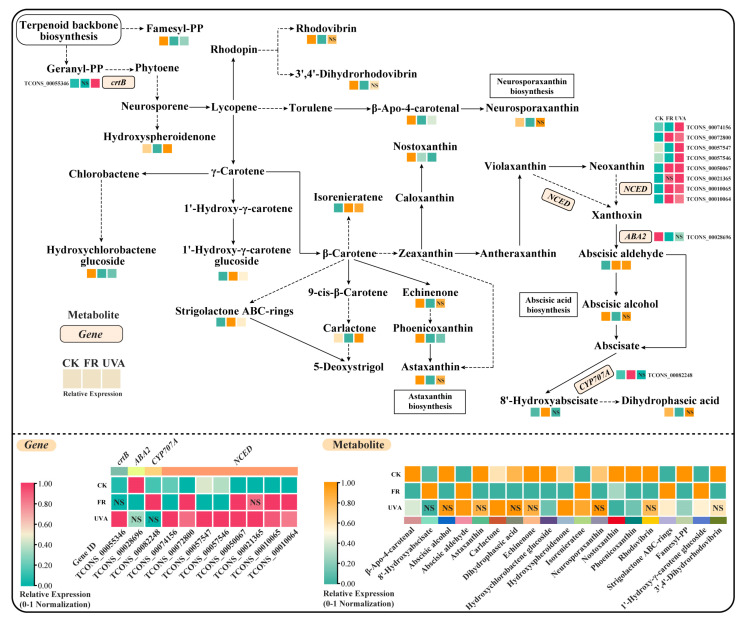
Differentially expressed genes (DEGs) and differentially accumulated metabolites (DAMs) in the carotenoid biosynthesis pathway under FR and UVA treatments. CK: The red-to-blue light ratio is maintained at 7:3 (7R3B); FR: 7R3B + far red light; UVA: 7R3B + ultraviolet-A light. Red or orange indicates a higher relative content, and green indicates a lower relative content. “NS” indicates that there is no significant difference in the relative content between FR and CK treatment or UVA and CK treatment.

**Figure 5:**
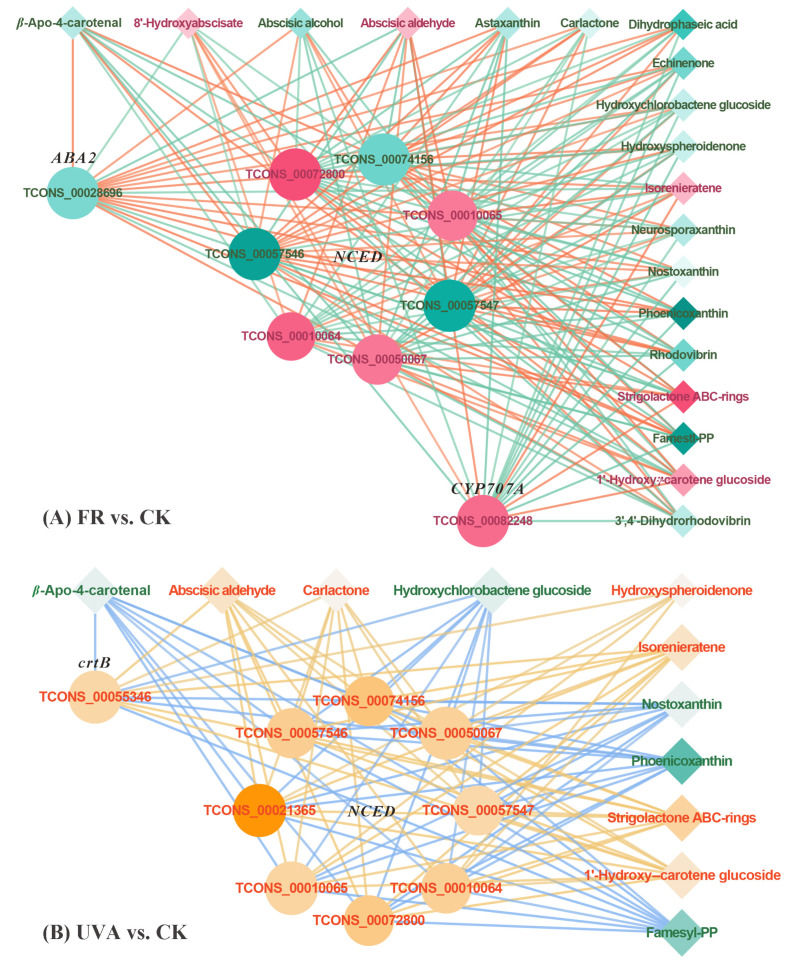
Correlation network diagram of differentially expressed genes (DEGs) and differentially accumulated metabolites (DAMs) in the carotenoid biosynthesis pathway under FR and UVA treatments. CK: The red-to-blue light ratio is maintained at 7:3 (7R3B); FR: 7R3B + far red light; UVA: 7R3B + ultraviolet-A light. Diamonds represent metabolites, and circles represent genes, with the correlation between them shown using straight lines. Positive correlations are represented by orange lines, while negative correlations are represented by green or blue lines, and the larger the absolute value of the correlation coefficient, the darker the color of the lines. The larger the gene or metabolite node, the larger the degree of enrichment. The node color represents the log2 fold change (log2 FC) in the expression difference in differentially expressed genes or metabolites, with colors leaning toward yellow or orange indicating log2 FC > 0, and colors leaning toward green or blue indicating log2 FC < 0.

Under the FR treatment, nine genes were differentially expressed (Figure 4), including five upregulated and four downregulated genes. In the FR vs. CK treatment group, the expression of *abscisic acid 8′-hydroxylase* (*CYP707A*) was upregulated, while *xanthoxin dehydrogenase* (*ABA2*) was downregulated (Figure 5A). Within *9-cis-epoxycarotenoid dioxygenase* (*NCED*), four genes were upregulated, and three genes were downregulated. In the FR vs. CK treatment group, 19 DAMs were identified, including 5 upregulated and 14 downregulated metabolites (Figure 4). The upregulated metabolites included isorenieratene and abscisic aldehyde, while the downregulated metabolites included neurosporaxanthin, astaxanthin, nostoxanthin, and β-Apo-4-carotenal (Figure 5A). The reduction in the levels of most metabolites in the carotenoid biosynthetic pathway under the FR treatment affected the carotenoid content.

Under the UVA treatment, nine genes exhibited differential expression (Figure 4), all of which were upregulated. In the UVA vs. CK treatment group (Figure 5B), the expression of *15-cis-phytoene synthase* (*crtB*) and *NCED* was upregulated. Furthermore, 11 metabolites showed differential accumulation, comprising 6 upregulated and 5 downregulated metabolites (Figure 4). The upregulated metabolites included carlactone and hydroxyspheroidenone, while conversely, the downregulated metabolites included nostoxanthin and β-Apo-4-carotenal (Figure 5B).

#### 2.5.2. Combined Analysis of Transcriptome and Metabolites in Plant Hormone Signaling Pathways Under FR Treatment

**Figure 6:**
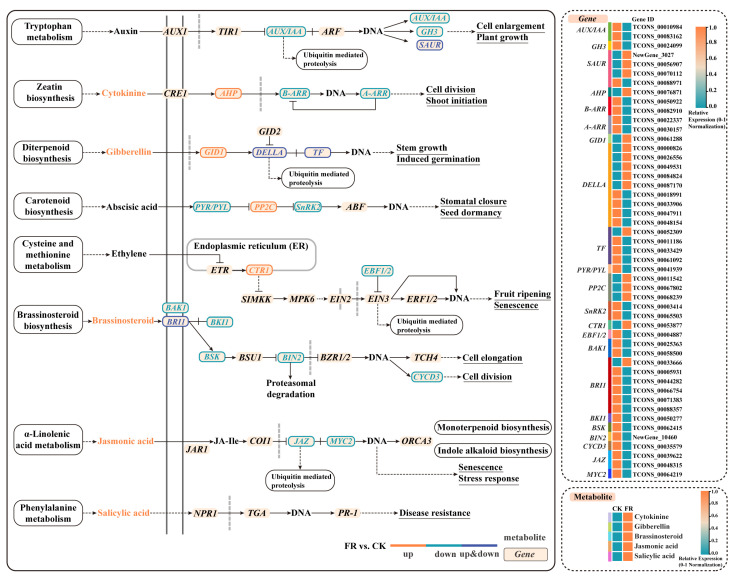
Differentially expressed genes (DEGs) and differentially accumulated metabolites (DAMs) in the plant hormone signaling pathway under the FR treatment. CK: The red-to-blue light ratio is maintained at 7:3 (7R3B); FR: 7R3B + far red light. The relative expression levels or concentrations of genes and metabolites are represented by orange and lake blue. Orange indicates a significantly higher relative amount and lake blue indicates a significantly lower relative amount.

**Figure 7:**
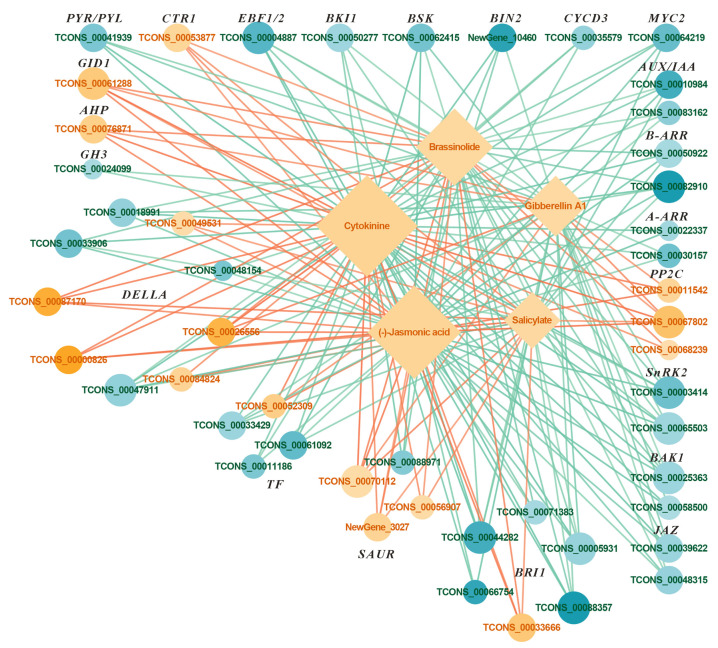
Correlation network diagram of differentially expressed genes (DEGs) and differentially accumulated metabolites (DAMs) in the plant hormone signal transduction pathway under the FR treatment. CK: The red-to-blue light ratio is maintained at 7:3 (7R3B); FR: 7R3B + far red light. Diamonds represent metabolites, and circles represent genes, with the correlation between them shown using straight lines. Positive correlations are represented by orange or yellow lines, while negative correlations are represented by green or blue lines, and the larger the absolute value of the correlation coefficient, the darker the color of the lines. The larger the gene or metabolite node, the larger the degree of enrichment. The node color represents the log2 fold change (log2 FC) in the expression difference in differentially expressed genes or metabolites, with colors leaning toward orange indicating log2 FC > 0, and colors leaning toward blue indicating log2 FC < 0.

Under the FR treatment, 49 genes were differentially expressed, including 16 upregulated and 33 downregulated genes (Figure 6 and Figure 7). Among the DEGs, one *histidine-containing phosphotransfer protein* (*AHP*), one *gibberellin receptor GID1* (*GID1*), three *protein phosphatase 2C* (*PP2C*), and one *serine/threonine-protein kinase CTR1* (*CTR1*) genes were upregulated; while two *auxin-responsive protein IAA* (*AUX/IAA*), one *auxin responsive GH3 gene family* (*GH3*), two *two-component response regulator ARR-B family* (*B-ARR*), two *two-component response regulator ARR-A family (A-ARR)*, one *abscisic acid receptor PYR/PYL family* (*PYR/PYL*), two *serine/threonine-protein kinase SRK2* (*SnRK2*), one *EIN3-binding F-box protein* (*EBF1/2*), two *brassinosteroid-insensitive 1-associated receptor kinase 1* (*BAK1*), one *BRI1 kinase inhibitor 1* (*BKI1*), one *BR-signaling kinase* (*BSK*), one *protein brassinosteroids-insensitive 2* (*BIN2*), one *cyclin D3, plant* (*CYCD3*), two *jasmonate ZIM domain-containing protein* (*JAZ*), and one *transcription factor MYC2* (*MYC2*) genes were downregulated (Figure 7). Among the DEGs, three *SAUR family protein* (*SAUR*) genes were upregulated, and one was downregulated; five *DELLA protein* (*DELLA*) genes were upregulated, and four were downregulated; one *phytochrome-interacting factor 4* (*TF*) gene was upregulated, and three were downregulated; and one *protein brassinosteroid-insensitive 1* (*BRI1*) gene was upregulated, and five were downregulated (Figure 7).

Five metabolites were differentially accumulated, all showing upregulation (Figure 6 and Figure 7). The DAMs include the upregulated ones: CTK, GA, BR, jasmonic acid (JA), and salicylic acid (SA) (Figure 6 and Figure 7). The analysis indicated that the FR treatment significantly affected the expression of five metabolites and their associated genes.

#### 2.5.3. Combined Analysis of Transcriptome and Metabolites in Flavonoid Biosynthesis Pathway Under UVA Treatment

**Figure 8:**
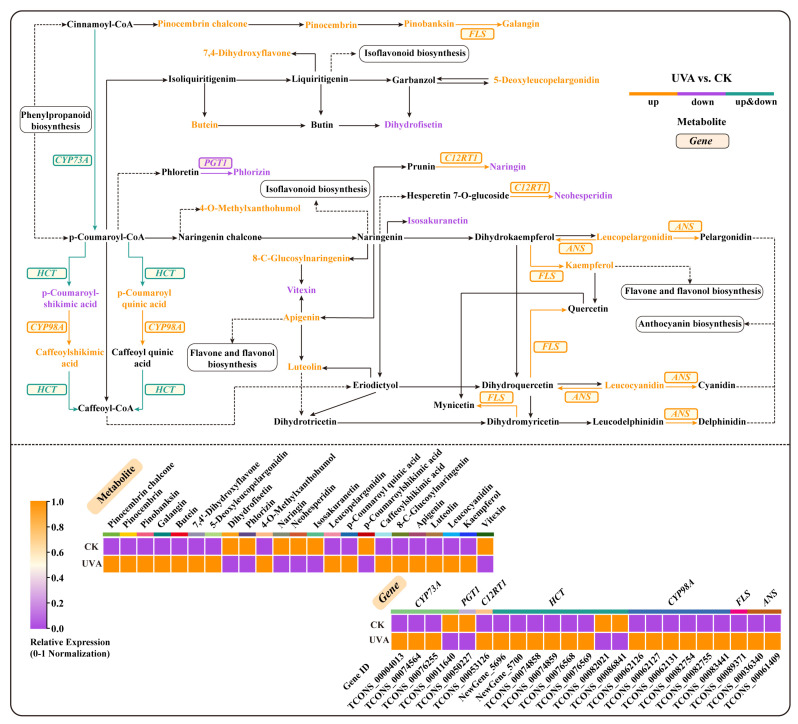
Differentially expressed genes (DEGs) and differentially accumulated metabolites (DAMs) in the flavonoid biosynthesis pathway under UVA treatment. CK: The red-to-blue light ratio is maintained at 7:3 (7R3B); UVA: 7R3B + ultraviolet-A light. The relative expression levels or concentrations of genes and metabolites are represented by orange and purple. Orange indicates a significantly higher relative amount and purple indicates a significantly lower relative amount.

**Figure 9:**
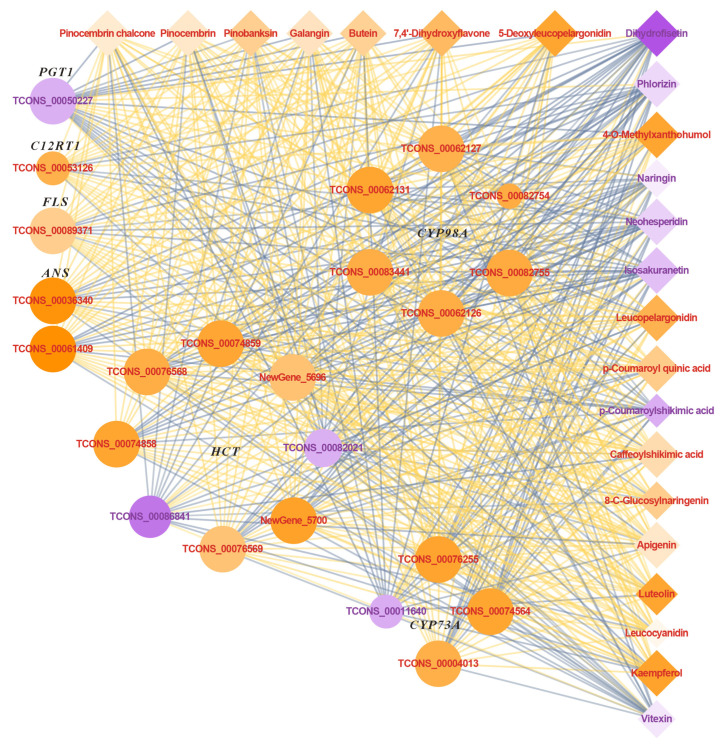
Correlation network diagram of differentially expressed genes (DEGs) and differentially accumulated metabolites (DAMs) in the flavonoid biosynthesis pathway under UVA treatment. CK: The red-to-blue light ratio is maintained at 7:3 (7R3B); UVA: 7R3B + ultraviolet-A light. Diamonds represent metabolites, and circles represent genes, with the correlation between them shown using straight lines. Positive correlations are represented by orange or yellow lines, while negative correlations are represented by green or blue lines, and the larger the absolute value of the correlation coefficient, the darker the color of the lines. The larger the gene or metabolite node, the larger the degree of enrichment. The node color represents the log2 fold change (log2 FC) in the expression difference in differentially expressed genes or metabolites, with colors leaning toward orange indicating log2 FC > 0, and colors leaning toward purple indicating log2 FC < 0.

By analyzing integrated transcriptomic and metabolomic data, we aimed to elucidate the flavonoid biosynthesis pathway in basil. Our findings revealed 23 DEGs, including 19 upregulated and 4 downregulated genes (Figure 8 and Figure 9). Among the DEGs, six *5-O-(4-coumaroyl)-D-quinate 3′-monooxygenase* (*CYP98A*), one *flavanone 7-O-glucoside 2″-O-beta-L-rhamnosyltransferase* (*C12RT1*), one *flavonol synthase* (*FLS*), and two *anthocyanidin synthase* (*ANS*) genes were upregulated, while one *phlorizin synthase* (*PGT1*) gene was downregulated (Figure 9). Among the DEGs, three *trans-cinnamate 4-monooxygenase* (*CYP73A*) were upregulated, and one was downregulated, while six *shikimate O-hydroxycinnamoyltransferase* (*HCT*) were upregulated, and two were downregulated.

Twenty-three differentially accumulated metabolites were identified, comprising 16 upregulated and 7 downregulated compounds (Figure 8 and Figure 9). Among the DAMs, the major upregulated compounds included luteolin, apigenin, kaempferol, and leucocyanidin, while the major downregulated compounds included naringin and neohesperidin (Figure 9).

### 2.6. Validation of RNA-Seq-Based DEG Results via Real-Time Quantitative PCR (qRT-PCR)

**Figure 10:**
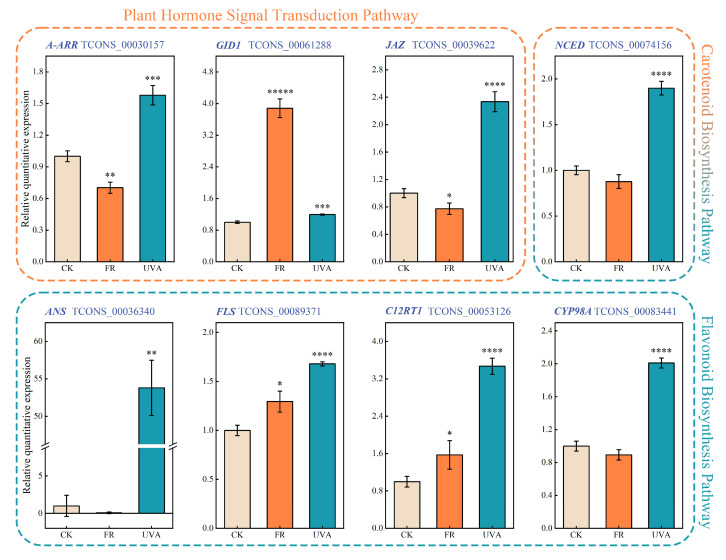
The effect of FR and UVA treatments on the transcription levels of related genes. CK: The red-to-blue light ratio is maintained at 7:3 (7R3B); FR: 7R3B + far red light; UVA: 7R3B + ultraviolet-A light. n = 3. Statistical significance is indicated as follows: *p* ≤ 0.05 (*), *p* ≤ 0.01 (**), *p* ≤ 0.001 (***), *p* ≤ 0.0001 (****), *p* ≤ 0.00001 (*****).

To further validate the effects of FR and UVA light on the expression levels of related genes in basil, a qRT-PCR analysis was conducted on a subset of genes involved in these pathways (Figure 10). In the carotenoid biosynthesis pathway, the UVA treatment significantly upregulated *NCED* expression. In the plant hormone signal transduction pathway, the FR treatment significantly downregulated the expression levels of *A-ARR* and *JAZ* while significantly upregulating the expression of *GID1* in response to a low R/FR light ratio. In the flavonoid biosynthesis pathway, the plant responded to high-intensity UV light by upregulating the expression of *ANS*, *FLS*, *C12RT1*, and *CYP98A* under the UVA treatment.

## 3. Discussions

This study provides novel insights into how FR light promotes basil growth and shade avoidance responses, while also elucidating the potential mechanisms underlying UVA-induced flavonoid biosynthesis, thereby further enhancing both yield and economic value.

### 3.1. Effects of FR and UVA Treatments on the Growth of Basil

Our study revealed that under the FR treatment, the plant height and stem diameter of basil significantly increased (Figure 1D,H), and the shoot fresh and dry weights were also significantly higher (Figure 1B,C). Supplementation with FR light has been shown to increase both the fresh and dry weight as well as the plant height of red leaf lettuce compared to white light [32]. Increasing the level of FR light exposure may potentially reduce the activity of photosensitive pigments, triggering a shading response in plants. This could lead to plants increasing their stem and petiole length to compete for more light resources, ultimately resulting in taller plant growth [33]. Vegetative growth and biomass accumulation in basil were enhanced by FR light, possibly through the simulation of shade conditions and activation of shade-avoidance responses. This suggests that manipulating FR light could be a practical strategy to increase the biomass yield in controlled environment agriculture for leafy herbs like basil.

Under the UVA treatment, stem diameter was significantly higher than that of the CK treatment (Figure 1E). Previous studies have shown that exposure to enhanced levels of UVA and UVA + UVB radiation can increase the biomass of laurel seedlings, with stem biomass increasing by 36% and 41%, respectively [34]. Other research has reported that supplementing different durations of UVA irradiation under white LED light positively influences the growth and development of kale. Under 6 and 12 h of the UVA treatment, plant height significantly increased, reaching 1.11 and 1.27 times that of the control, respectively. Additionally, a 12-h UVA treatment resulted in a 10.07% increase in the stem diameter [35]. Consistently with previous studies, this study also found that the UVA treatment had a positive effect on stem growth in basil, leading to an increase in stem diameter.

### 3.2. Effects of FR and UVA Treatments on Chlorophyll Content of Basil

In our study, the FR treatment led to a significant reduction in the chlorophyll content in basil leaves (Table 1). Similarly, substituting a portion of photosynthetically active radiation with FR light has been shown to significantly reduce the relative chlorophyll content in basil leaves [36]. Related research suggests that the continuous addition of FR light during plant growth downregulates the photosynthetic capacity of leaves, as indicated by a reduction in chlorophyll content [37]. It can be seen that long-term FR light treatment is not conducive to the accumulation of chlorophyll in basil leaves.

In barley (*Hordeum vulgare* L.), exposure to UVA radiation has been reported to increase total chlorophyll content by 13% compared to plants grown without UVA [38]. In lettuce, replacing part of the blue light with UVA significantly reduced the chlorophyll *a* content, while having no significant effect on chlorophyll *b* [39]. Other studies have shown that varying UVA intensities do not significantly influence chlorophyll content in lettuce [40]. Similarly, in our study, the UVA treatment had no significant effect on the chlorophyll content of basil (Table 1). These findings suggest that the effect of UVA on chlorophyll accumulation varies among different plant species.

### 3.3. Effects of FR and UVA Treatments on Carotenoid Content and the Carotenoid Biosynthesis Pathway in Basil

In this study, FR and UVA treatments exhibited different effects on carotenoid content in basil. DEGs and DAMs showed distinct patterns within the carotenoid biosynthesis pathway under FR and UVA treatments, indicating the necessity of further investigation into this pathway.

**Figure 11:**
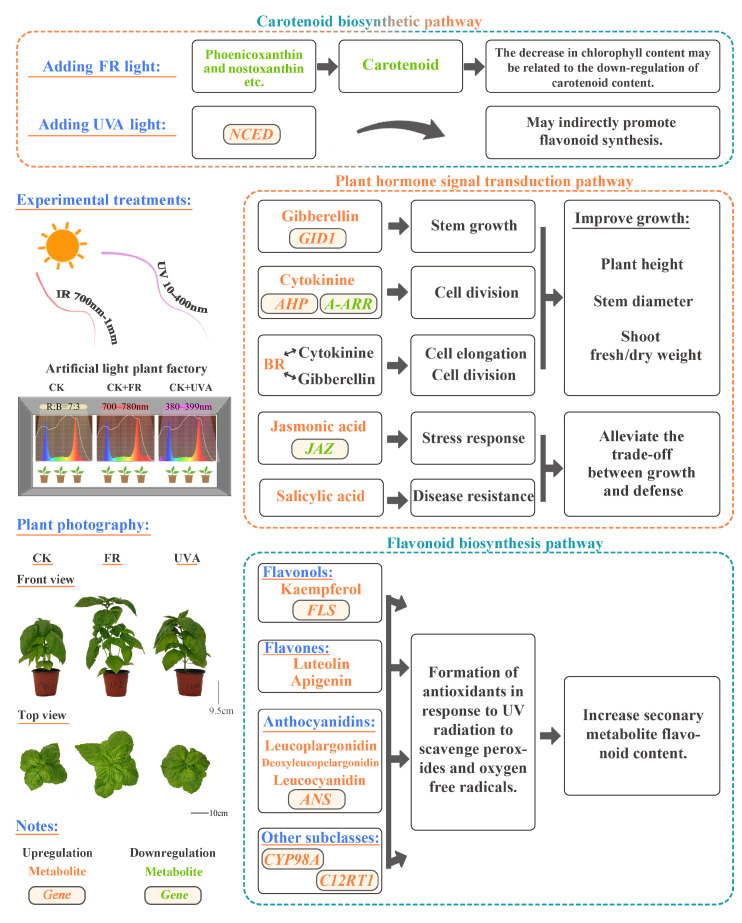
Effects of added FR and UVA light on basil growth, chlorophyll and carotenoid content, transcriptome, and metabolome. CK: The red-to-blue light ratio is maintained at 7:3 (7R3B); FR: 7R3B + far red light; UVA: 7R3B + ultraviolet-A light.

Previous studies have shown that supplemental FR light can increase the yield of red and yellow sweet pepper by 9% and 19%, respectively, while decreasing the carotenoid content by 26% and 9% [41]. Similarly, the addition of FR light has been reported to reduce the carotenoid content in lettuce by 12–16% [42]. Other findings suggest that increased chlorophyll levels are associated with elevated β-carotene levels in plants [43]. In this study, the FR treatment downregulated key metabolites in the carotenoid biosynthesis pathway, thereby reducing the carotenoid content (Table 1, Figure 5A). Moreover, the decrease in the carotenoid content may be unfavorable to the accumulation of chlorophyll, ultimately leading to a significant decrease in the chlorophyll content of basil (Figure 11).

The effects of UV radiation on the carotenoid content in plants remain inconclusive. Exposure to both UVA and UVB radiation has been shown to increase the carotenoid content in eight varieties of green leaf lettuce [44]. Conversely, the carotenoid levels in another eight varieties of red leaf lettuce decreased upon supplementation with ultraviolet radiation [44]. The underlying reasons for the emergence of such contradictions remain unclear. However, according to relevant research reports, the induction of carotenoids may be associated with the dosage and duration of UV radiation exposure, as well as the specific properties of certain carotenoids (such as lutein and β-carotene) [45,46]. In this study, the UVA treatment did not significantly affect carotenoid content in basil (Table 1). However, in the sweet basil cultivar “Rubin”, the addition of UV radiation has been shown to increase the carotenoid content [47]. The differences in results may be partly explained by the variation in basil cultivars, as “Rubin” sweet basil is a red-leaf variety, whereas the cultivar used in this study is a green-leaf type. Furthermore, the carotenoid content of different basil varieties is influenced differently by UVA radiation of varying wavelengths. A study found contrasting results in different basil varieties following UVA treatments at four different wavelengths (343 nm, 366 nm, 386 nm, 402 nm). Specifically, UVA radiation at 402nm wavelength enhanced the carotenoid content in green leaf basil, while treatments at 402nm and 386nm wavelengths reduced the carotenoid content in red leaf basil [48]. The effect of UVA on the carotenoid content of basil may be related to the leaf color of basil varieties and the wavelength of UVA light.

Studies have shown that UVC treatment upregulates *NCED* expression, which in turn increases ABA levels. ABA and its related transcription factors may be involved in regulating flavonoid synthesis driven by UVC radiation [49]. In this study, the upregulation of *NCED* expression in the carotenoid biosynthesis pathway under the UVA treatment may indirectly promote the synthesis of flavonoids (Figure 11).

### 3.4. Effects of FR Treatment on the Hormone Signaling Transduction Pathway in Basil

In this study, the FR treatment upregulated GA levels and *GID1* gene expression in the plant hormone signaling pathway, and significantly affected the expression of *DELLA* genes (Figure 6 and Figure 7). The extension of the photoperiod using FR light may promote stem elongation by enhancing the plant’s responsiveness to endogenous GA_1_ [50]. Upon binding with GA, *GID1* forms a GID1-GA complex that interacts with DELLA proteins. DELLA proteins inhibit plant growth in the absence of GA; however, when bound to the GID1-GA complex, they are tagged for degradation. This degradation releases the GA signaling pathway, thereby promoting plant growth and development [51]. The stability of DELLA proteins is regulated not only by GA but also by ethylene and IAA [52,53], which may be a key integrative protein in response to different environmental and endogenous hormone regulations.

The FR treatment significantly increased the CTK levels in basil and upregulated the expression of the *AHP* gene (Figure 6 and Figure 7). The upregulation of CTK provides additional activation signals, while the increased expression of *AHP* ensures the efficient transmission and amplification of these signals, thereby promoting plant cell division. Although the precise role of CTK in mediating the effects of light on stem elongation remains unclear, it likely exerts its influence through interactions with other hormones [54,55]. Evidence suggests a negative interaction between GA and CTK [56]. Both GA and CTK can independently promote stem elongation in soybean plants [57]; however, when applied together, CTK inhibits the action of GA, primarily by reducing internode number, stem diameter, and overall plant size. Under light conditions, CTK interacts with the ethylene signaling pathway and selectively upregulates the synthesis of ethylene and IAA, as compared to under dark conditions [58].

Our study also revealed that the level of BR under the FR treatment was significantly higher (Figure 6 and Figure 7). BR may promote localized stem elongation by altering the mechanical properties of the cell wall, such as facilitating cell wall loosening [59]. Related research has found that compared to red light, the stimulatory effect of low R/FR light ratios on GA_1_ is associated with BR [60]. BR can enhance the action of IAA under a low R/FR light ratio, promoting cell elongation and division, and helping plants overcome shading issues [61]. The exogenous application of BR significantly increased the CTK levels in wheat seedlings [62]. The increase in BR may also have a positive effect on the increase in CTK content in plant growth.

A low R/FR ratio shifts the carbon resource allocation towards growth at the expense of defense, thereby increasing plant susceptibility to pathogen attack [63]. Previous studies have shown that JA [64] and SA [65] play important roles in plant defense, stress responses, and disease resistance. Elevated levels of these hormones help prepare the plant for potential biotic threats [66]. Upon the perception of damage or stress signals, JA and its active derivatives (such as JA-Ile) accumulate, leading to the degradation of JAZs. This degradation releases the repression on the transcription factor *MYC2*, thereby activating the expression of JA-responsive genes [67].

FR light-promoted basil growth may thus be achieved not only via the upregulation of GA biosynthesis and signaling (*GID1* upregulation and *DELLA* degradation) but also through complex hormonal crosstalk involving BR, CTK, and IAA, which together orchestrate a coordinated developmental response (Figure 11). These findings provide molecular evidence for the hormonal basis of FR-induced shade avoidance in basil. Additionally, in the plant hormone signal transduction pathway, the FR treatment enhanced the plant defense and stress resistance of basil by downregulating the negative regulatory factor *JAZ* and upregulating the levels of JA and SA metabolites. Changes in the expression of defense hormones and related genes may partially alleviate the trade-off between growth and defense under low R/FR conditions (Figure 11).

### 3.5. Effects of UVA Treatment on the Flavonoid Biosynthetic Pathway in Basil

This study found that the UVA treatment significantly affected the gene expression and metabolite accumulation in the flavonoid biosynthesis pathway of basil (Figure 8 and Figure 9). Flavonoids are divided into subclasses according to the functional groups present on the C ring, which include anthocyanidins, flavanols, flavones, flavonols, flavanones, and isoflavonoids [68].

Among the upregulated metabolites under the UVA treatment (Figure 8 and Figure 9), kaempferol is one of the most common flavonol glycosides in the flavonol class. In addition, the UVA treatment upregulated the expression of *FLS*. Transgenic Arabidopsis plants expressing maize *FLS_1_* accumulated high levels of flavonols in their leaves [69]. These transgenic plants exhibited significantly higher levels of kaempferol, approximately 3.8 times higher than the control. As a result, they showed reduced UVB-induced DNA damage under high-intensity UV irradiation, along with less damage to the photosynthetic electron transport chain and membranes [69]. In this study, the UVA treatment led to the upregulation of *FLS* expression, which facilitated the accumulation of kaempferol, and could potentially help mitigate DNA damage induced by UVA light in basil.

Flavones are the largest group of flavonoid compounds present in plants. In this study, the levels of luteolin and apigenin were found to be upregulated under the UVA treatment (Figure 8 and Figure 9). The primary flavones, luteolin and apigenin, have been reported to enhance plant tolerance to UV radiation. Under high UVB exposure, the ratio of luteolin to apigenin increases, suggesting that luteolin may function more effectively than apigenin as an antioxidant in scavenging reactive oxygen species [70]. Moreover, when *Ligustrum vulgare* is grown under supplemental UV light, levels of luteolin derivatives tend to increase further [71]. High apigenin accumulation has been associated with reduced sensitivity to UVB-induced damage, providing protective effects against cellular damage, including that to DNA, lipids, membranes, and photosynthetic pigments [72]. In this study, the UVA treatment may have enhanced the antioxidant capacity of basil by upregulating the levels of luteolin and apigenin.

Anthocyanins are among the most widely distributed flavonoids in plants. This study found that under the UVA treatment, the expression of *ANS* was upregulated, accompanied by increased levels of anthocyanin-related metabolites (leucocyanidin, 5-deoxyleucopelargonidin, and cis-3,4-leucopelargonidin) (Figure 8). Relevant studies have demonstrated that supplemental UV irradiation enhances the accumulation of higher levels of anthocyanins in tomatoes within a short period [73,74]. Research has shown that anthocyanin-containing plants exhibit greater efficacy in preventing pyrimidine dimer formation following UV irradiation compared to anthocyanin-deficient counterparts, thereby attaining UV shielding effects [75]. Furthermore, ANS participates in the synthesis of anthocyanins. Related studies have found that UVB can induce the expression of *Malus domestica MYB transcription factor A* (*MdMYBA*) in apple peel, whereby *MdMYBA* can specifically bind to the promoter of ANS, inducing the accumulation of anthocyanins in both apple buds and tomato fruits [76]. In this study, *ANS* expression was upregulated, which in turn promoted the accumulation of anthocyanin-related metabolites, which was beneficial in increasing the ability of basil to cope with UVA.

In this study, the UVA treatment upregulated the expression of the *CYP98A* and *C12RT1* genes (Figure 8). Previous research has demonstrated that enhanced UV exposure in plants promotes the upregulation of *CYP98A* expression, which in turn leads to an increase in flavonoid compounds [77]. Experimental evidence indicates that *C12RT1* promotes the synthesis of secondary metabolites, such as flavonoids [78]. However, the specific role of *C12RT1* in flavonoid biosynthesis remains unclear and warrants further investigation. The coordinated upregulation of *CYP98A* and *C12RT1* expression established a metabolic foundation for enhanced flavonoid biosynthesis in basil.

UVA light can be an effective inducer for increasing the flavonoid content in basil (Figure 11), which has potential significance for improving the nutritional and medicinal value of basil through spectrum optimization in cultivation systems.

## 4. Materials and Methods

### 4.1. Plant Material and Growth Conditions

Experiments were conducted in a plant factory at the National Engineering Research Center of Protected Agriculture in Chongming District, Shanghai, to investigate the effects of FR light and UVA light treatments on biomass, chlorophyll content, and transcriptional and metabolic responses in basil. The basil seeds were provided by Beijing Fengming Yashi Technology Development Co., Ltd. (Beijing, China). Seeds were sown and seedlings were raised in an LED artificial light plant factory. Germinated seeds were planted in 128-cell trays. The temperature in the plant factory is maintained at 25 °C during the day and 23 °C at night, with a relative humidity of 75 ± 10%. The photoperiod is set to 13 h of light and 11 h of darkness. Plants were grown under a photosynthetic photon flux density (PPFD) of approximately 221 μmol m^−2^ s^−1^ during the cultivation period. Twenty days after sowing (average plant height 3.4 cm), the seedlings in the plug trays were transplanted into pots about 11 cm in diameter and 9.5 cm in height, which were filled with coir substrate. Subsequently, various light treatments were conducted within the controlled environment of a plant factory. Each treatment consisted of 10 plants with three biological replications.

The electrical conductivity (EC) value of the nutrient solution is (2.0 ± 0.2) dS m^−1^, and the pH is approximately 5.5 [79]. In addition, we provide the main ingredients of the nutrient solution (Supplementary Materials: Table S4), and irrigation was performed as required. The seedlings were irrigated with tidal irrigation, and the pots were kept soaked in the nutrient solution for 30 min. The excess nutrient solution was then drained to ensure there was enough nutrient solution in the pots during the entire growth period. Initial growth measurements were taken the day before initiating the light treatments. Harvesting and sampling were conducted uniformly after 33 days.

### 4.2. Experimental Treatments

This experiment included three treatments. The control group (CK) was set with a 7:3 photosynthetic photon flux (PPF) ratio of red to blue light. The FR treatment consisted of a 13% far-red light supplementation relative to the CK treatment. The UVA treatment involved the addition of 2% ultraviolet-A light relative to the CK treatment. Plants were grown under each of these three different spectra (Supplementary Materials: Figure S2) until the end of the experiment. The PPF and PPFD settings for each treatment group are outlined in the table below (Table 2). The PPF data were provided by Shanghai Sansi Electronic Engineering Co., Ltd., Shanghai, China. The PPFD values under different treatments were measured using the Lighting Passport (ALP-01, Asensetek, Taiwan, China). The Lighting Passport was placed at the initial height of the plants, approximately 25 cm from the light source.

### 4.3. Project Measurement

#### 4.3.1. Measurement of Growth Indicators

Each treatment randomly selected 5 plants for measurements of plant height, canopy width, stem diameter, maximum leaf length, and maximum leaf width. Plant height, canopy width, maximum leaf length, and maximum leaf width were measured using a measuring tape, while stem diameter was measured using a vernier caliper. Growth indicators were continuously measured throughout the experimental period, and line charts were plotted to observe their dynamic changes. Shoot fresh weight was measured using electronic scales. The plants were then dried in an oven at 120 °C until reaching a constant weight, after which their dry weight was recorded. Shoot dry weight was determined using an electronic balance.

#### 4.3.2. Determination of Chlorophyll and Carotenoid Contents

Chlorophyll and carotenoid extraction was conducted using the classical organic solvent method. A 0.1 g sample of fresh leaf tissue was weighed and placed into a mortar, followed by the addition of 10 mL of anhydrous ethanol as the extraction solvent. The sample was thoroughly ground with a pestle until a homogeneous green suspension was obtained. The suspension was then transferred into a 1.5 mL centrifuge tube and centrifuged at 12,000 rpm for 10 min. The supernatant was collected as the chlorophyll extract. To ensure extraction efficiency, each sample was extracted three times, and the supernatants were combined. The absorbance of the chlorophyll and carotenoid extract was measured using a microplate reader (Model: Infinite 200Pro, Manufacturer: Tecan, Switzerland). The absorbance was recorded at wavelengths of 664 nm, 647 nm, and 470nm after the appropriate dilution of the extract. Subsequently, the contents of chlorophyll *a*, chlorophyll *b*, and carotenoids were calculated using the following formulas. The total chlorophyll content was determined as the sum of chlorophyll *a* and chlorophyll *b*.

$$C_{a}=13.95A_{664}-6.88A_{647}$$

$$C_{b}=24.96A_{647}-7.32A_{664}$$

$$C_{x\;c}=\frac{1000A_{470}-2.05C_{a}-114.8C_{b}}{245}$$

In the formula, *C_a_* and *C_b_* represent the concentrations of chlorophyll *a* and chlorophyll *b*, respectively; *C_x__c_* denotes the total concentration of carotenoids. *A664*, *A647*, and *A470* correspond to the absorbance values of the chloroplast pigment extract at wavelengths of 664 nm, 647 nm, and 470 nm, respectively.

$$C(\mathrm{mg}/g)=\frac{C\cdot V\cdot N}{W\cdot 1000}$$

In the formula, *C* represents the pigment content (mg g^−1^); *V* is the volume of the extraction solution (10 mL); *N* is the dilution factor (1); *W* is the fresh or dry weight of the sample (0.1 g); and 1000 is the conversion factor from milliliters to liters (1 L = 1000 mL).

#### 4.3.3. Transcriptome Sequencing and Data Analysis

(1) RNA extraction: Total RNA from plants was extracted using the RNA prep Pure Plant Kit (Tiangen, Beijing, China). RNA concentration and purity were measured using a NanoDrop 2000 (Thermo Fisher Scientific, Wilmington, DE, USA). RNA integrity was assessed using the RNA Nano 6000 Assay Kit with the Agilent Bioanalyzer 2100 system (Agilent Technologies, Santa Clara, CA, USA).
(2) RNA quantification and qualification: RNA concentration and purity were measured using NanoDrop 2000. RNA integrity was assessed using the RNA Nano 6000 Assay Kit of the Agilent Bioanalyzer 2100 system.
(3) Library preparation for transcriptome sequencing: A total amount of 1 μg RNA per sample was used as the input material for the RNA sample preparations. Sequencing libraries were generated using Hieff NGS Ultima Dual-mode mRNA Library Prep Kit for Illumina (Yeasen Biotechnology (Shanghai) Co., Ltd., Shanghai, China) following the manufacturer’s recommendations, and index codes were added to attribute sequences to each sample.
(4) Sequencing: The libraries were sequenced on an Illumina NovaSeq platform to generate 150 bp paired-end reads, according to the manufacturer’s instructions.
(5) Data analysis: The raw reads were further processed with a bioinformatic pipeline tool—the BMKCloud (https://www.biocloud.net (accessed on 2 November 2023)) online platform.

Quality control: Raw data (raw reads) of fastq format were first processed through in-house Perl scripts. In this step, clean data (clean reads) were obtained by removing reads containing adapter, reads containing ploy-N, and low-quality reads from raw data. At the same time, the Q20, Q30, GC-content, and sequence duplication levels of the clean data were calculated. All the downstream analyses were based on clean data with high quality.

Reads mapping to the reference genome: The adaptor sequences and low-quality sequence reads were removed from the data sets. Raw sequences were transformed into clean reads after data processing. These clean reads were then mapped to the reference genome sequence. Only reads with a perfect match or one mismatch were further analyzed and annotated based on the reference genome. The Hisat2 (v 2.0.4) software tools (Center for Computational Biology, Johns Hopkins University, Baltimore, MD, USA) were used to map with the reference genome.

Gene functional annotation: Gene function was annotated based on the following databases: NCBI non-redundant protein sequences (Nr); protein family (Pfam); clusters of orthologous groups of proteins (KOG/COG); a manually annotated and reviewed protein sequence database (Swiss-Prot); KEGG Ortholog database (KO); gene ontology (GO).

Differential expression analysis: A differential expression analysis of two samples was performed using edgeR. An FDR < 0.01 and a fold change ≥ 2 were set as the threshold values for a significantly differential expression.

Kyoto Encyclopedia of Genes and Genomes (KEGG) pathway enrichment analysis: We used the KOBAS database (http://bioinfo.org/kobas/ (accessed on 12 November 2023)) and the clusterProfiler (v 4.4.4) software tool (Bioconductor, New York, NY, USA) to test the statistical enrichment of the differential expression of genes in KEGG pathways.

#### 4.3.4. Metabolome Sequencing and Data Analysis

(1) Metabolite Extraction. The LC/MS system for metabolomics analysis comprises a Waters Acquity I-Class PLUS ultra-high performance liquid tandem Waters Xevo G2-XS QTof (Waters, Milford, MA, USA) high-resolution mass spectrometer. The column used is purchased from Waters Acquity UPLC HSS T3 column (1.8 μm 2.1 × 100 mm).
(2) LC-MS/MS Analysis. The Waters Xevo G2-XS QTof high-resolution mass spectrometer can be used to collect primary and secondary mass spectrometry data in the MSe mode under the control of the acquisition software program (MassLynx v 4.2, Waters, Milford, MA, USA).
(3) Data Preprocessing and Annotation. The raw data collected using MassLynx v 4.2 (Waters, Milford, MA, USA) is processed using the Progenesis QI software v 3.0 (Waters, Newcastle upon Tyne, UK) for peak extraction, peak alignment, and other data processing operations, based on the Progenesis QI software online METLIN database and Biomark’s self-built library (https://www.biocloud.net (accessed on 5 November 2023)) for identification, and at the same time, the theoretical fragment identification and mass deviation are all within 100ppm [80].
(4) Data Analysis. The identified compounds are searched for classification and pathway information in the KEGG (http://www.genome.jp/kegg/ (accessed on 11 November 2023)), HMDB (https://hmdb.ca/ (accessed on 13 November 2023)), and lipidmaps (https://lipidmaps.org/ (accessed on 16 November 2023)) databases. According to the grouping information, the difference multiples were calculated and compared, and a *t*-test was used to calculate the difference in significance of each compound’s *p* value. The VIP value of the model was calculated using multiple cross-validation. The method of combining the difference multiple, the *p* value, and the VIP value of the OPLS-DA model was adopted to screen the differential metabolites. The screening criteria are a fold change > 1, *p*-value < 0.05, and VIP > 1. The differential metabolites of the KEGG pathway enrichment significance were calculated using the hypergeometric distribution test [81]. The raw data were further processed using the bioinformatics analysis platform BMKCloud (https://www.biocloud.net (accessed on 21 November 2023)) to obtain the analytical results related to differentially accumulated metabolites.

#### 4.3.5. Correlation Network Diagram of Differentially Expressed Genes (DEGs) and Differentially Accumulated Metabolites (DAMs)

The correlations between all genes and metabolites were calculated based on the Pearson correlation (Pearson) method for each differential subgroup, and the data were preprocessed using the z-value transformation method before calculating the correlations; the data were then screened according to the correlation coefficient (CC) and the *p* value of the correlations, and the thresholds for screening were |CC| > 0.80 and CCP < 0.05. The differential metabolites and differential genes of each subgroup were screened based on the results of the correlation analysis, combined with the results of the correlation analysis: 0.80 and CCP < 0.05. The screening was then based on the differential metabolites and differential genes of each subgroup, combined with the results of the correlation analysis. The differential genes and differential metabolites screened by correlation were selected according to the pathway for mapping.

#### 4.3.6. Quantitative Real-Time PCR (qRT-PCR) Validation

The total RNA was extracted from plant leaves using AM1912 RNAqueous^®^ Kit (Thermo Fisher Scientific, Waltham, MA, USA). The reverse transcription reaction was performed using the GeneAmp PCR System 9700 (Applied Biosystems, Foster City, CA, USA). A real-time PCR analysis was conducted on a LightCycler 480 II Real-Time PCR Instrument (Roche, Basel, CH) with reactions incubated in a 384-well plate, and each sample was analyzed in triplicate. Primer sequences and relative expression levels are provided in Supplementary Materials (Supplementary Materials: Table S5). Actin (actin depolymerizing factor2, partial) was used as the reference gene to normalize the expression levels of target genes, and relative expression was calculated using the 2^(−ΔΔCt)^ method [82].

### 4.4. Data Analysis

The experimental data were organized using Microsoft Office Excel 2016. An analysis of variance (ANOVA) was performed using SPSS 27.0 (IBM Corp., Armonk, NY, USA), with comparisons of means conducted via a one-way ANOVA, showing significant differences at a 95% confidence interval. The results are expressed using mean ± standard deviation. Graphs were created using OriginPro 2022 (OriginLab Corporation, Northampton, MA, USA), Photoshop 2024 (Adobe Systems, San Jose, CA, USA), and Cytoscape v 3.10.3 (Cytoscape Consortium, San Diego, CA, USA).

## 5. Conclusions

In summary, the addition of FR light can promote basil growth by influencing gene expression and metabolite accumulation in the plant hormone signaling pathway, although it reduces the content of photosynthetic pigments. The addition of UVA can enhance flavonoid accumulation by affecting the gene expression and metabolite accumulation in the flavonoid biosynthesis pathway.

While this study focused on the individual effects of FR and UVA light, it remains unclear whether these two light spectra interact synergistically or antagonistically. Further investigation is needed to determine how they collectively influence plant metabolic networks and morphological development under combined light conditions.

## Figures and Tables

**Table 1:** Chlorophyll and carotenoid content.

Treatment	Chlorophyll a (mg g^−1^)	Chlorophyll b (mg g^−1^)	Chlorophyll (mg g^−1^)	Carotenoid (mg g^−1^)
CK	0.907 ± 0.003 ^a^	0.323 ± 0.002 ^b^	1.23 ± 0.004 ^a^	0.17 ± 0.001 ^a^
FR	0.81 ± 0.023 ^b^	0.285 ± 0.007 ^c^	1.095 ± 0.029 ^b^	0.147 ± 0.005 ^b^
UVA	0.914 ± 0.017 ^a^	0.332 ± 0.004 ^a^	1.247 ± 0.02 ^a^	0.165 ± 0.004 ^a^

Note: CK: The red-to-blue light ratio is maintained at 7:3 (7R3B); FR: 7R3B + far red light; UVA: 7R3B + ultraviolet-A light. Different letters indicate significant differences at α = 0.05 based on the least significant difference (LSD) test (n = 3).

**Table 2:** PPF and PPFD settings under different treatments.

Treatment	Photosynthetic Photon Flux (PPF) (μmol s^−1^)						Photosynthetic Photon Flux Density (PPFD) (μmol m^−2^ s^−1^)					
	R	B	G	FR	UVA	Total PPF	R	B	G	FR	UVA	Total PPFD
CK	529	225	94	0.06	0.05	848.1	125.4	67.9	28.4	5.57	0.13	221.6
FR	530	224	93	106	0.02	953	125.4	68	28.5	27.7	0.09	221.9
UVA	531	232	93	0.11	17	873.1	125.2	70.7	28.2	5.4	1.85	224.1

Note: CK: the red-to-blue light ratio is maintained at 7:3 (7R3B); FR: 7R3B + far red light; UVA: 7R3B + ultraviolet-A light. Wavelength range of light: R (red light: 600–699 nm); B (blue light: 400–499 nm); G (green light: 500–599 nm); FR (far-red light: 700–780 nm); UVA (ultraviolet-A light: 380–399 nm).

## Data Availability

Data are contained within the article and Supplementary Materials.

## Supplementary Materials

The supporting information can be downloaded at: https://www.mdpi.com/article/10.3390/ijms26083758/s1.
