# Supplementary material for: Transcriptomic and Metabolomic Insights into Plant Hormone Modulation and Secondary Metabolite Accumulation in Basil Under Far-Red and Ultraviolet-A Light
Source: Int J Mol Sci. 2025 Apr 16;26(8):3758. doi: 10.3390/ijms26083758 (PMC12027552; doi:10.3390/ijms26083758)
Supplement: Supplementary file 1 [file ijms-26-03758-s001.zip › ijms-3519875-supplementary.pdf]

Table S1. The clean reads for each sample

Sample ID	Clean reads	Clean bases	GC(%)	Q30(%)
CK-1	20747666	6219090819	47.54	97.73
CK-2	23110503	6925965834	47.63	97.65
CK-3	23428741	7020642330	47.74	97.51
FR-1	23371229	7004802041	47.57	97.43
FR-2	21063632	6313639116	47.7	97.89
FR-3	21347858	6398869352	47.7	98
UVA-1	20361984	6103767406	47.75	98.16
UVA-2	21784113	6529039315	47.69	97.88
UVA-3	22412166	6717117281	47.58	97.68

Note: Sample ID: Sample analysis number; Clean reads: Total number of pair-end reads in the Clean Data; Clean bases: Total base count in the Clean Data; GC: GC content of the Clean Data, i.e., the percentage of G and C bases in the Clean Data relative to the total base count; Q30: Percentage of bases in the Clean Data with a quality score greater than or equal to 30.

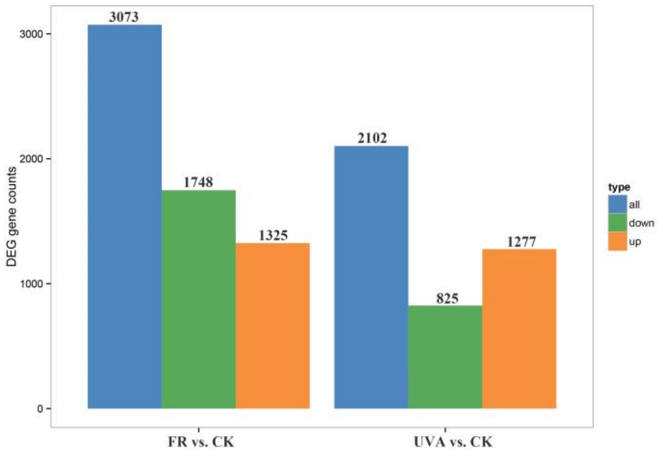

Figure S1. Bar chart of differentially expressed genes.

Table S2. Statistical table of differentially expressed gene counts annotated in different databases

DEG Set	COG	GO	KEGG	KOG	NR	Pfam	Swiss-Prot
FR vs. CK	512	1,391	1,085	748	1,734	1,332	1,206
UVA vs. CK	406	941	785	543	1,146	895	832

Note: DEG Set: name of differentially expressed gene set; the second to last columns indicate the number of differentially expressed genes annotated by each functional database.

Table S3. Statistical table of differential metabolites

Group	Diff num	UP num	Down num

FR vs. CK	1708	918	790
UVA vs. CK	1586	964	622

Note: Group: differential metabolite grouping information; Diff num: number of significantly differential metabolites; Up num: number of upregulated metabolites; Down num: number of downregulated metabolites.

Table S4. The content of various elements in the nutrient solution for irrigation

Element	Fe	Mn	B	Zn	Cu	Mo
Content ($g \cdot L^{-1}$)	4.563×10^{-4}	1.281×10^{-4}	9.660×10^{-5}	1.218×10^{-4}	2.065×10^{-5}	1.491×10^{-5}
Element	Ca	Mg	K	S	N	P
Content ($g \cdot L^{-1}$)	7.816×10^{-2}	2.020×10^{-2}	6.711×10^{-2}	3.224×10^{-2}	6.850×10^{-2}	1.183×10^{-2}

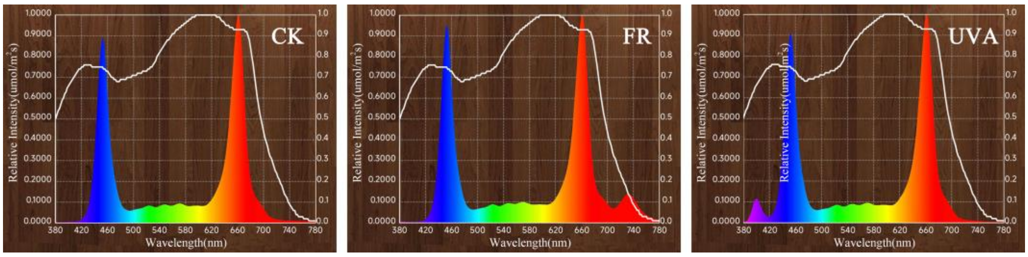

Figure S2. Spectrograms of CK (A), FR (B) and UVA (C) under three different treatments.

Table S5. Details on the design of primers used for quantitative real-time PCR (qRT-PCR)

Num	Name	Gene Symbol	Forward primer (5≥3)	Reverse primer (5≥3)	Product length(bp)	Tm (℃)
1	*Actin*	TCONS_00004099	GTTATTGTGGAGAAGCTCGG	TATCTGCACTCATCAGCAGGA	80	60
2	*GID1*	TCONS_00061288	CCGAGTTCAAGTTCCTCAC	TGTTTCTCCGGCAGGTAT	121	60
3	*JAZ*	TCONS_00039622	GCCGTATCAAGCAACGAAG	CTTGGCTGAATTTGGAGCG	84	60
4	*A-ARR*	TCONS_00030157	CAAGAATCACAAGATGCTTGG	CATCATGTGCTCCTGTAGT	95	60
5	*FLS*	TCONS_00089371	GCTAGGACCCTACTTATGCAG	CAATATCGATGACCGGAATGG	83	60
6	*ANS*	TCONS_00036340	TTCCTGAATTATCTCCCGCT	ACCACTTCTCTACTGTACTCAC	101	60
7	*CYP98A*	TCONS_00083441	GTGGTGACATTCATGAACACT	TAATCATCCACACCAATGCG	89	60
8	*C12RT1*	TCONS_00053126	CTCATGGCCACATCTTCC	AGATTGATGGGTGTTGAGC	94	60
9	*NCED*	TCONS_00074156	CATATCACCTGCGAAATGCT	AATATGGTGGAAGAAGATTCGG	91	60
